# Supplementary figures and images for: Impact of community-based health insurance in low- and middle-income countries: A systematic review and meta-analysis
Source: PLoS One. 2023 Jun 27;18(6):e0287600. doi: 10.1371/journal.pone.0287600 (PMC10298805; doi:10.1371/journal.pone.0287600)

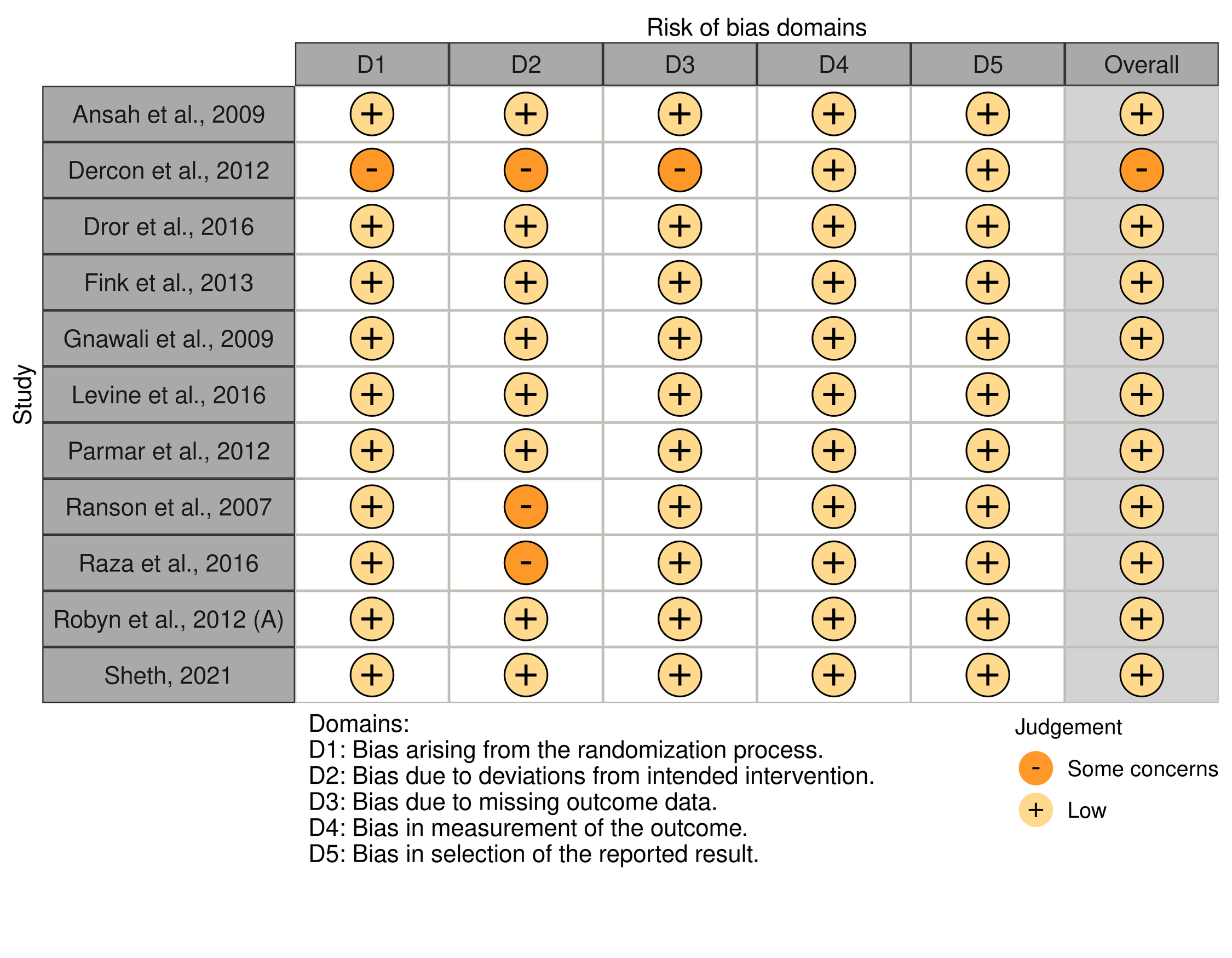

Supplement: S1 Fig — (TIF) [file pone.0287600.s001.tif]

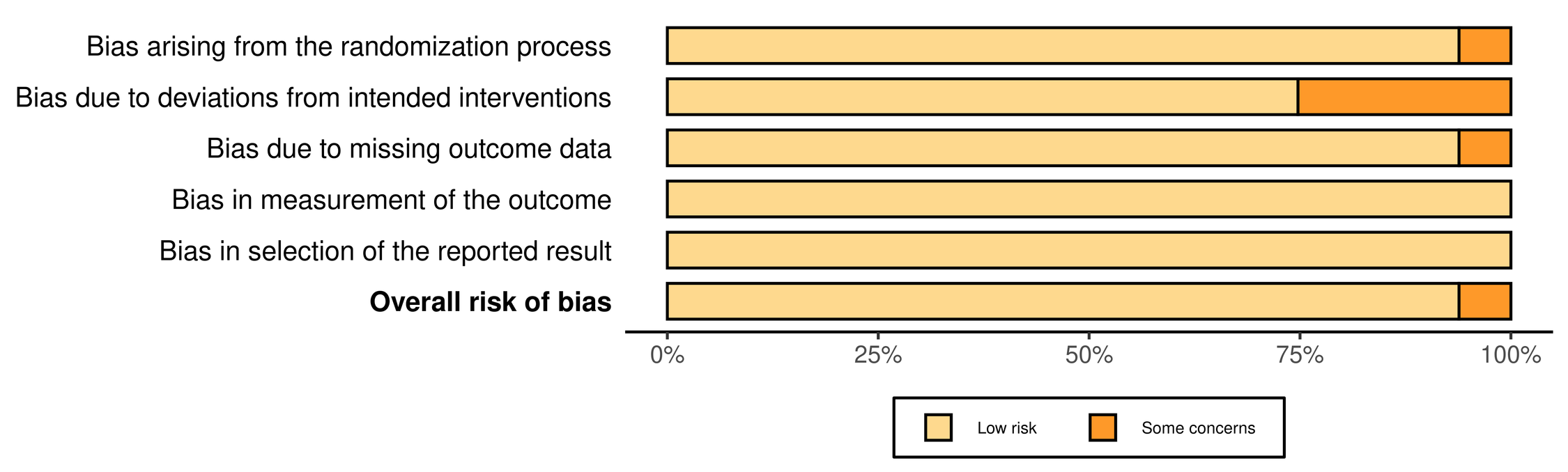

Supplement: S2 Fig — (TIF) [file pone.0287600.s002.tif]

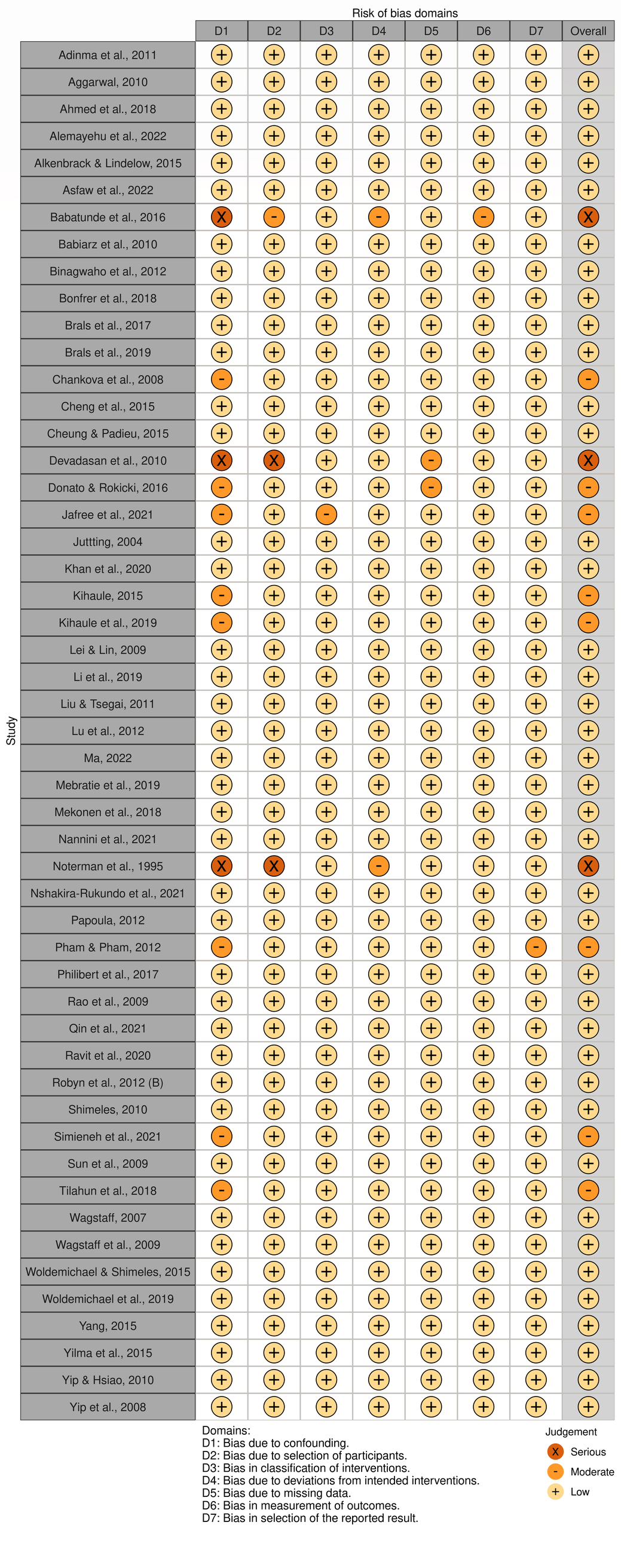

Supplement: S3 Fig — (TIF) [file pone.0287600.s003.tif]

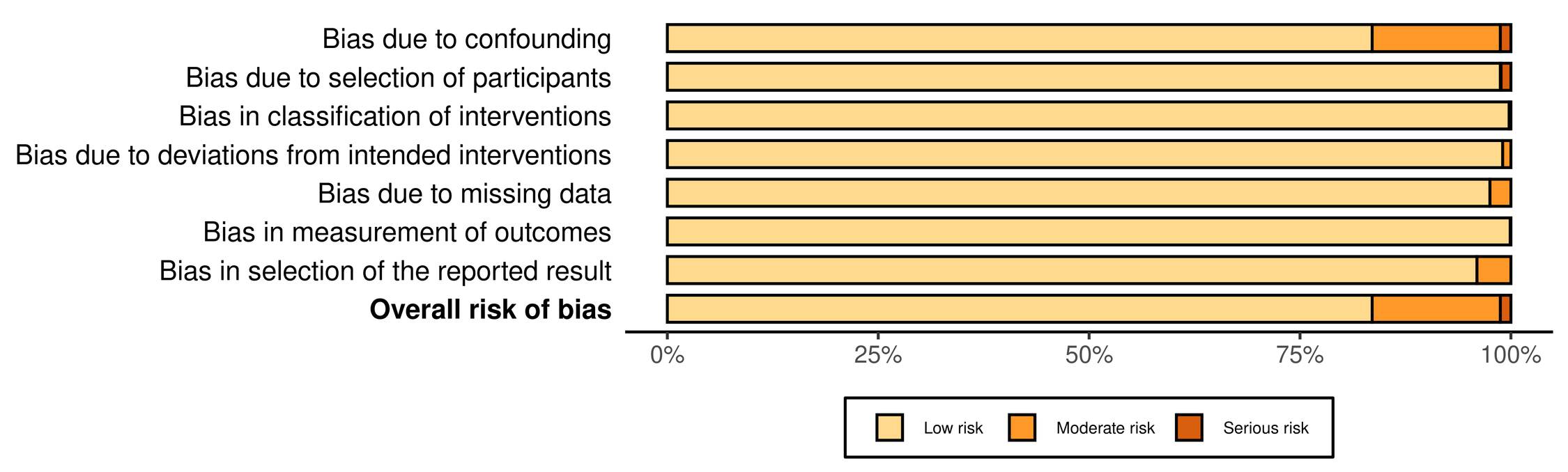

Supplement: S4 Fig — (TIF) [file pone.0287600.s004.tif]
