# Supplementary material for: Impact of community-based health insurance in low- and middle-income countries: A systematic review and meta-analysis
Source: PLoS One. 2023 Jun 27;18(6):e0287600. doi: 10.1371/journal.pone.0287600 (PMC10298805; doi:10.1371/journal.pone.0287600)
Supplement: S1 Text — (DOCX) [file pone.0287600.s005.docx]

**Supplement 1:** Search Strategy

**PubMed**

Search date: 04 October 2022

Results: 7,471

Search: Search: ((((((((community based health insurance) OR (community-based health insurance)) OR (community health fund)) OR (mutual health insurance)) OR (micro health insurance)) OR (hospital prepayment scheme)) OR (community-based prepayment scheme)) AND (((((((((((((((catastrophic expen*) OR (catastrophic health payment*)) OR (catastrophic health expen*)) OR (catastrophic health expenditure)) OR (financial risk protection)) OR (financial protection)) OR (financial protection)) OR (financial catastrophe)) OR (health payment induced poverty)) OR (catastrophic out of pocket payments)) OR (catastrophic out of pocket expenditure)) OR (out of pocket payments)) OR (out of pocket expenditure)) OR (healthcare utilization)) OR (healthcare access))) AND (((((((((developing countr*) OR (less developed countr*)) OR (less developed countr*)) OR (least developed countr*)) OR (underdeveloped nations)) OR (third world countries)) OR (low income countries)) OR (middle income countries)) OR ("Afghanistan" OR "Albania" OR "Algeria" OR "American Samoa" OR "Angola" OR "Argentina" OR "Armenia" OR "Azerbaijan" OR "Bangladesh" OR "Belarus" OR "Belize" OR "Benin" OR "Bhutan" OR "Bolivia" OR "Bosnia and Herzegovina" OR "Botswana" OR "Brazil" OR "Bulgaria" OR "Burkina Faso" OR "Burundi" OR "Cabo Verde" OR "Cambodia" OR "Cameroon" OR "Central African Republic" OR "Chad" OR "China" OR "Colombia" OR "Comoros" OR "Congo Democratic Republic" OR "Congo Republic" OR "Costa Rica" OR "Côte d'Ivoire" OR "Cuba" OR "Djibouti" OR "Dominica" OR "Dominican Republic" OR "Ecuador" OR "Egypt" OR "El Salvador" OR "Equatorial Guinea" OR "Eritrea" OR "Eswatini" OR "Ethiopia" OR "Fiji" OR "Gabon" OR "The Gambia" OR "Georgia" OR "Ghana" OR "Grenada" OR "Guatemala" OR "Guinea" OR "Guinea-Bissau" OR "Guyana" OR "Haiti" OR "Honduras" OR "India" OR "Indonesia" OR "Iran, Islamic Republic" OR "Iraq" OR "Jamaica" OR "Jordan" OR "Kazakhstan" OR "Kenya" OR "Kiribati" OR "Korea, Democratic People's Republic" OR "Kosovo" OR "Kyrgyz Republic" OR "Lao PDR" OR "Lebanon" OR "Lesotho" OR "Liberia" OR "Libya" OR "Madagascar" OR "Malawi" OR "Malaysia" OR "Maldives" OR "Mali" OR "Marshall Islands" OR "Mauritania" OR "Mexico" OR "Micronesia" OR "Moldova" OR "Mongolia" OR "Montenegro" OR "Morocco" OR "Mozambique" OR "Myanmar" OR "Namibia" OR "Nepal" OR "Nicaragua" OR "Niger" OR "Nigeria" OR "North Macedonia" OR "Pakistan" OR "Papua New Guinea" OR "Paraguay" OR "Peru" OR "Philippines" OR "Russian Federation" OR "Rwanda" OR "Samoa" OR "São Tomé and Principe" OR "Senegal" OR "Serbia" OR "Sierra Leone" OR "Solomon Islands" OR "Somalia" OR "South Africa" OR "South Sudan" OR "Sri Lanka" OR "St. Lucia" OR "St. Vincent and the Grenadines" OR "Sudan" OR "Suriname" OR "Syrian Arab Republic" OR "Tajikistan" OR "Tanzania" OR "Thailand" OR "Timor-Leste" OR "Togo" OR "Tonga" OR "Tunisia" OR "Turkey" OR "Turkmenistan" OR "Tuvalu" OR "Uganda" OR "Ukraine" OR "Uzbekistan" OR "Vanuatu" OR "Venezuela" OR "Vietnam" OR "West Bank and Gaza" OR "Yemen Republic" OR "Zambia" OR "Zimbabwe")) Sort by: Most Recent

("community based health insurance"[MeSH Terms] OR ("community-based"[All Fields] AND "health"[All Fields] AND "insurance"[All Fields]) OR "community based health insurance"[All Fields] OR ("community"[All Fields] AND "based"[All Fields] AND "health"[All Fields] AND "insurance"[All Fields]) OR "community based health insurance"[All Fields] OR ("community based health insurance"[MeSH Terms] OR ("community-based"[All Fields] AND "health"[All Fields] AND "insurance"[All Fields]) OR "community based health insurance"[All Fields] OR ("community"[All Fields] AND "based"[All Fields] AND "health"[All Fields] AND "insurance"[All Fields]) OR "community based health insurance"[All Fields]) OR (("public health"[MeSH Terms] OR ("public"[All Fields] AND "health"[All Fields]) OR "public health"[All Fields] OR ("community"[All Fields] AND "health"[All Fields]) OR "community health"[All Fields]) AND ("financial management"[MeSH Terms] OR ("financial"[All Fields] AND "management"[All Fields]) OR "financial management"[All Fields] OR "fund"[All Fields])) OR ("community based health insurance"[MeSH Terms] OR ("community-based"[All Fields] AND "health"[All Fields] AND "insurance"[All Fields]) OR "community based health insurance"[All Fields] OR ("mutual"[All Fields] AND "health"[All Fields] AND "insurance"[All Fields]) OR "mutual health insurance"[All Fields]) OR ("community based health insurance"[MeSH Terms] OR ("community-based"[All Fields] AND "health"[All Fields] AND "insurance"[All Fields]) OR "community based health insurance"[All Fields] OR ("micro"[All Fields] AND "health"[All Fields] AND "insurance"[All Fields]) OR "micro health insurance"[All Fields]) OR (("hospital s"[All Fields] OR "hospitalisation"[All Fields] OR "hospitalization"[MeSH Terms] OR "hospitalization"[All Fields] OR "hospitalising"[All Fields] OR "hospitality"[All Fields] OR "hospitalisations"[All Fields] OR "hospitalised"[All Fields] OR "hospitalizations"[All Fields] OR "hospitalized"[All Fields] OR "hospitalize"[All Fields] OR "hospitalizing"[All Fields] OR "hospitals"[MeSH Terms] OR "hospitals"[All Fields] OR "hospital"[All Fields]) AND ("prepayment"[All Fields] OR "prepayments"[All Fields]) AND ("scheme"[All Fields] OR "scheme s"[All Fields] OR "schemes"[All Fields])) OR ("community-based"[All Fields] AND ("prepayment"[All Fields] OR "prepayments"[All Fields]) AND ("scheme"[All Fields] OR "scheme s"[All Fields] OR "schemes"[All Fields]))) AND ((("catastrophe"[All Fields] OR "catastrophes"[All Fields] OR "catastrophic"[All Fields] OR "catastrophically"[All Fields]) AND "expen*"[All Fields]) OR (("catastrophe"[All Fields] OR "catastrophes"[All Fields] OR "catastrophic"[All Fields] OR "catastrophically"[All Fields]) AND ("health"[MeSH Terms] OR "health"[All Fields] OR "health s"[All Fields] OR "healthful"[All Fields] OR "healthfulness"[All Fields] OR "healths"[All Fields]) AND "payment*"[All Fields]) OR (("catastrophe"[All Fields] OR "catastrophes"[All Fields] OR "catastrophic"[All Fields] OR "catastrophically"[All Fields]) AND ("health"[MeSH Terms] OR "health"[All Fields] OR "health s"[All Fields] OR "healthful"[All Fields] OR "healthfulness"[All Fields] OR "healths"[All Fields]) AND "expen*"[All Fields]) OR (("catastrophe"[All Fields] OR "catastrophes"[All Fields] OR "catastrophic"[All Fields] OR "catastrophically"[All Fields]) AND ("health expenditures"[MeSH Terms] OR ("health"[All Fields] AND "expenditures"[All Fields]) OR "health expenditures"[All Fields] OR ("health"[All Fields] AND "expenditure"[All Fields]) OR "health expenditure"[All Fields])) OR (("economics"[MeSH Terms] OR "economics"[All Fields] OR "financial"[All Fields] OR "financially"[All Fields] OR "financials"[All Fields] OR "financier"[All Fields] OR "financiers"[All Fields]) AND ("risk"[MeSH Terms] OR "risk"[All Fields]) AND ("protect"[All Fields] OR "protected"[All Fields] OR "protecting"[All Fields] OR "protection"[All Fields] OR "protections"[All Fields] OR "protective agents"[Pharmacological Action] OR "protective agents"[MeSH Terms] OR ("protective"[All Fields] AND "agents"[All Fields]) OR "protective agents"[All Fields] OR "protectant"[All Fields] OR "protectants"[All Fields] OR "protective"[All Fields] OR "protectively"[All Fields] OR "protectiveness"[All Fields] OR "protectives"[All Fields] OR "protects"[All Fields])) OR (("economics"[MeSH Terms] OR "economics"[All Fields] OR "financial"[All Fields] OR "financially"[All Fields] OR "financials"[All Fields] OR "financier"[All Fields] OR "financiers"[All Fields]) AND ("protect"[All Fields] OR "protected"[All Fields] OR "protecting"[All Fields] OR "protection"[All Fields] OR "protections"[All Fields] OR "protective agents"[Pharmacological Action] OR "protective agents"[MeSH Terms] OR ("protective"[All Fields] AND "agents"[All Fields]) OR "protective agents"[All Fields] OR "protectant"[All Fields] OR "protectants"[All Fields] OR "protective"[All Fields] OR "protectively"[All Fields] OR "protectiveness"[All Fields] OR "protectives"[All Fields] OR "protects"[All Fields])) OR (("economics"[MeSH Terms] OR "economics"[All Fields] OR "financial"[All Fields] OR "financially"[All Fields] OR "financials"[All Fields] OR "financier"[All Fields] OR "financiers"[All Fields]) AND ("protect"[All Fields] OR "protected"[All Fields] OR "protecting"[All Fields] OR "protection"[All Fields] OR "protections"[All Fields] OR "protective agents"[Pharmacological Action] OR "protective agents"[MeSH Terms] OR ("protective"[All Fields] AND "agents"[All Fields]) OR "protective agents"[All Fields] OR "protectant"[All Fields] OR "protectants"[All Fields] OR "protective"[All Fields] OR "protectively"[All Fields] OR "protectiveness"[All Fields] OR "protectives"[All Fields] OR "protects"[All Fields])) OR (("economics"[MeSH Terms] OR "economics"[All Fields] OR "financial"[All Fields] OR "financially"[All Fields] OR "financials"[All Fields] OR "financier"[All Fields] OR "financiers"[All Fields]) AND ("catastrophe"[All Fields] OR "catastrophes"[All Fields] OR "catastrophic"[All Fields] OR "catastrophically"[All Fields])) OR (("health"[MeSH Terms] OR "health"[All Fields] OR "health s"[All Fields] OR "healthful"[All Fields] OR "healthfulness"[All Fields] OR "healths"[All Fields]) AND ("compensation and redress"[MeSH Terms] OR ("compensation"[All Fields] AND "redress"[All Fields]) OR "compensation and redress"[All Fields] OR "payment"[All Fields] OR "payments"[All Fields]) AND ("induce"[All Fields] OR "induced"[All Fields] OR "inducer"[All Fields] OR "inducers"[All Fields] OR "induces"[All Fields] OR "inducibilities"[All Fields] OR "inducibility"[All Fields] OR "inducible"[All Fields] OR "inducing"[All Fields]) AND ("poverty"[MeSH Terms] OR "poverty"[All Fields] OR "poverty s"[All Fields])) OR (("catastrophe"[All Fields] OR "catastrophes"[All Fields] OR "catastrophic"[All Fields] OR "catastrophically"[All Fields]) AND ("health expenditures"[MeSH Terms] OR ("health"[All Fields] AND "expenditures"[All Fields]) OR "health expenditures"[All Fields] OR ("out"[All Fields] AND "pocket"[All Fields] AND "payments"[All Fields]) OR "out of pocket payments"[All Fields])) OR (("catastrophe"[All Fields] OR "catastrophes"[All Fields] OR "catastrophic"[All Fields] OR "catastrophically"[All Fields]) AND ("health expenditures"[MeSH Terms] OR ("health"[All Fields] AND "expenditures"[All Fields]) OR "health expenditures"[All Fields] OR ("out"[All Fields] AND "pocket"[All Fields] AND "expenditure"[All Fields]) OR "out of pocket expenditure"[All Fields])) OR ("health expenditures"[MeSH Terms] OR ("health"[All Fields] AND "expenditures"[All Fields]) OR "health expenditures"[All Fields] OR ("out"[All Fields] AND "pocket"[All Fields] AND "payments"[All Fields]) OR "out of pocket payments"[All Fields]) OR ("health expenditures"[MeSH Terms] OR ("health"[All Fields] AND "expenditures"[All Fields]) OR "health expenditures"[All Fields] OR ("out"[All Fields] AND "pocket"[All Fields] AND "expenditure"[All Fields]) OR "out of pocket expenditure"[All Fields]) OR (("delivery of health care"[MeSH Terms] OR ("delivery"[All Fields] AND "health"[All Fields] AND "care"[All Fields]) OR "delivery of health care"[All Fields] OR "healthcare"[All Fields] OR "healthcare s"[All Fields] OR "healthcares"[All Fields]) AND ("statistics and numerical data"[MeSH Subheading] OR ("statistics"[All Fields] AND "numerical"[All Fields] AND "data"[All Fields]) OR "statistics and numerical data"[All Fields] OR "utilization"[All Fields] OR "utilisation"[All Fields] OR "utilisations"[All Fields] OR "utilise"[All Fields] OR "utilised"[All Fields] OR "utilises"[All Fields] OR "utilising"[All Fields] OR "utilities"[All Fields] OR "utility"[All Fields] OR "utilizations"[All Fields] OR "utilize"[All Fields] OR "utilized"[All Fields] OR "utilizer"[All Fields] OR "utilizers"[All Fields] OR "utilizes"[All Fields] OR "utilizing"[All Fields])) OR (("delivery of health care"[MeSH Terms] OR ("delivery"[All Fields] AND "health"[All Fields] AND "care"[All Fields]) OR "delivery of health care"[All Fields] OR "healthcare"[All Fields] OR "healthcare s"[All Fields] OR "healthcares"[All Fields]) AND ("access"[All Fields] OR "accessed"[All Fields] OR "accesses"[All Fields] OR "accessibilities"[All Fields] OR "accessibility"[All Fields] OR "accessible"[All Fields] OR "accessing"[All Fields]))) AND ((("develop"[All Fields] OR "develope"[All Fields] OR "developed"[All Fields] OR "developer"[All Fields] OR "developer s"[All Fields] OR "developers"[All Fields] OR "developing"[All Fields] OR "developments"[All Fields] OR "develops"[All Fields] OR "growth and development"[MeSH Subheading] OR ("growth"[All Fields] AND "development"[All Fields]) OR "growth and development"[All Fields] OR "development"[All Fields]) AND "countr*"[All Fields]) OR ("less"[All Fields] AND ("develop"[All Fields] OR "develope"[All Fields] OR "developed"[All Fields] OR "developer"[All Fields] OR "developer s"[All Fields] OR "developers"[All Fields] OR "developing"[All Fields] OR "developments"[All Fields] OR "develops"[All Fields] OR "growth and development"[MeSH Subheading] OR ("growth"[All Fields] AND "development"[All Fields]) OR "growth and development"[All Fields] OR "development"[All Fields]) AND "countr*"[All Fields]) OR ("less"[All Fields] AND ("develop"[All Fields] OR "develope"[All Fields] OR "developed"[All Fields] OR "developer"[All Fields] OR "developer s"[All Fields] OR "developers"[All Fields] OR "developing"[All Fields] OR "developments"[All Fields] OR "develops"[All Fields] OR "growth and development"[MeSH Subheading] OR ("growth"[All Fields] AND "development"[All Fields]) OR "growth and development"[All Fields] OR "development"[All Fields]) AND "countr*"[All Fields]) OR ("least"[All Fields] AND ("develop"[All Fields] OR "develope"[All Fields] OR "developed"[All Fields] OR "developer"[All Fields] OR "developer s"[All Fields] OR "developers"[All Fields] OR "developing"[All Fields] OR "developments"[All Fields] OR "develops"[All Fields] OR "growth and development"[MeSH Subheading] OR ("growth"[All Fields] AND "development"[All Fields]) OR "growth and development"[All Fields] OR "development"[All Fields]) AND "countr*"[All Fields]) OR (("underdeveloped"[All Fields] OR "underdevelopment"[All Fields]) AND ("ethnicity"[MeSH Terms] OR "ethnicity"[All Fields] OR "nationalities"[All Fields] OR "nationality"[All Fields] OR "federal government"[MeSH Terms] OR ("federal"[All Fields] AND "government"[All Fields]) OR "federal government"[All Fields] OR "national"[All Fields] OR "nation"[All Fields] OR "nation s"[All Fields] OR "nationalism"[All Fields] OR "nationalisms"[All Fields] OR "nationalization"[All Fields] OR "nationalized"[All Fields] OR "nationally"[All Fields] OR "nationals"[All Fields] OR "nations"[All Fields] OR "nations s"[All Fields])) OR ("developing countries"[MeSH Terms] OR ("developing"[All Fields] AND "countries"[All Fields]) OR "developing countries"[All Fields] OR ("third"[All Fields] AND "world"[All Fields] AND "countries"[All Fields]) OR "third world countries"[All Fields]) OR (("poverty"[MeSH Terms] OR "poverty"[All Fields] OR ("low"[All Fields] AND "income"[All Fields]) OR "low income"[All Fields]) AND ("countries"[All Fields] OR "country"[All Fields] OR "country s"[All Fields] OR "countrys"[All Fields])) OR (("middle"[All Fields] OR "middles"[All Fields]) AND ("income"[MeSH Terms] OR "income"[All Fields] OR "incomes"[All Fields] OR "income s"[All Fields]) AND ("countries"[All Fields] OR "country"[All Fields] OR "country s"[All Fields] OR "countrys"[All Fields])) OR ("Afghanistan"[All Fields] OR "Albania"[All Fields] OR "Algeria"[All Fields] OR "American Samoa"[All Fields] OR "Angola"[All Fields] OR "Argentina"[All Fields] OR "Armenia"[All Fields] OR "Azerbaijan"[All Fields] OR "Bangladesh"[All Fields] OR "Belarus"[All Fields] OR "Belize"[All Fields] OR "Benin"[All Fields] OR "Bhutan"[All Fields] OR "Bolivia"[All Fields] OR "Bosnia and Herzegovina"[All Fields] OR "Botswana"[All Fields] OR "Brazil"[All Fields] OR "Bulgaria"[All Fields] OR "Burkina Faso"[All Fields] OR "Burundi"[All Fields] OR "Cabo Verde"[All Fields] OR "Cambodia"[All Fields] OR "Cameroon"[All Fields] OR "Central African Republic"[All Fields] OR "Chad"[All Fields] OR "China"[All Fields] OR "Colombia"[All Fields] OR "Comoros"[All Fields] OR "Congo Democratic Republic"[All Fields] OR "Congo Republic"[All Fields] OR "Costa Rica"[All Fields] OR "Cote d'Ivoire"[All Fields] OR "Cuba"[All Fields] OR "Djibouti"[All Fields] OR "Dominica"[All Fields] OR "Dominican Republic"[All Fields] OR "Ecuador"[All Fields] OR "Egypt"[All Fields] OR "El Salvador"[All Fields] OR "Equatorial Guinea"[All Fields] OR "Eritrea"[All Fields] OR "Eswatini"[All Fields] OR "Ethiopia"[All Fields] OR "Fiji"[All Fields] OR "Gabon"[All Fields] OR "The Gambia"[All Fields] OR "Georgia"[All Fields] OR "Ghana"[All Fields] OR "Grenada"[All Fields] OR "Guatemala"[All Fields] OR "Guinea"[All Fields] OR "Guinea-Bissau"[All Fields] OR "Guyana"[All Fields] OR "Haiti"[All Fields] OR "Honduras"[All Fields] OR "India"[All Fields] OR "Indonesia"[All Fields] OR "iran islamic republic"[All Fields] OR "Iraq"[All Fields] OR "Jamaica"[All Fields] OR "Jordan"[All Fields] OR "Kazakhstan"[All Fields] OR "Kenya"[All Fields] OR "Kiribati"[All Fields] OR "korea democratic people s republic"[All Fields] OR "Kosovo"[All Fields] OR "Kyrgyz Republic"[All Fields] OR "Lao PDR"[All Fields] OR "Lebanon"[All Fields] OR "Lesotho"[All Fields] OR "Liberia"[All Fields] OR "Libya"[All Fields] OR "Madagascar"[All Fields] OR "Malawi"[All Fields] OR "Malaysia"[All Fields] OR "Maldives"[All Fields] OR "Mali"[All Fields] OR "Marshall Islands"[All Fields] OR "Mauritania"[All Fields] OR "Mexico"[All Fields] OR "Micronesia"[All Fields] OR "Moldova"[All Fields] OR "Mongolia"[All Fields] OR "Montenegro"[All Fields] OR "Morocco"[All Fields] OR "Mozambique"[All Fields] OR "Myanmar"[All Fields] OR "Namibia"[All Fields] OR "Nepal"[All Fields] OR "Nicaragua"[All Fields] OR "Niger"[All Fields] OR "Nigeria"[All Fields] OR "North Macedonia"[All Fields] OR "Pakistan"[All Fields] OR "Papua New Guinea"[All Fields] OR "Paraguay"[All Fields] OR "Peru"[All Fields] OR "Philippines"[All Fields] OR "Russian Federation"[All Fields] OR "Rwanda"[All Fields] OR "Samoa"[All Fields] OR "Sao Tome and Principe"[All Fields] OR "Senegal"[All Fields] OR "Serbia"[All Fields] OR "Sierra Leone"[All Fields] OR "Solomon Islands"[All Fields] OR "Somalia"[All Fields] OR "South Africa"[All Fields] OR "South Sudan"[All Fields] OR "Sri Lanka"[All Fields] OR "st lucia"[All Fields] OR "st vincent and the grenadines"[All Fields] OR "Sudan"[All Fields] OR "Suriname"[All Fields] OR "Syrian Arab Republic"[All Fields] OR "Tajikistan"[All Fields] OR "Tanzania"[All Fields] OR "Thailand"[All Fields] OR "Timor-Leste"[All Fields] OR "Togo"[All Fields] OR "Tonga"[All Fields] OR "Tunisia"[All Fields] OR "Turkey"[All Fields] OR "Turkmenistan"[All Fields] OR "Tuvalu"[All Fields] OR "Uganda"[All Fields] OR "Ukraine"[All Fields] OR "Uzbekistan"[All Fields] OR "Vanuatu"[All Fields] OR "Venezuela"[All Fields] OR "Vietnam"[All Fields] OR "West Bank and Gaza"[All Fields] OR "Yemen Republic"[All Fields] OR "Zambia"[All Fields] OR "Zimbabwe"[All Fields]))

**EconLit**

Search date: 07 October 2022

Result: 80

( AB community based health insurance OR AB mutual health insurance OR AB micro health insurance OR AB prepayment scheme ) AND ( ("Afghanistan" OR "Albania" OR "Algeria" OR "American Samoa" OR "Angola" OR "Argentina" OR "Armenia" OR "Azerbaijan" OR "Bangladesh" OR "Belarus" OR "Belize" OR "Benin" OR "Bhutan" OR "Bolivia" OR "Bosnia and Herzegovina" OR "Botswana" OR "Brazil" OR "Bulgaria" OR "Burkina Faso" OR "Burundi" OR "Cabo Verde" OR "Cambodia" OR "Cameroon" OR "Central African Republic" OR "Chad" OR "China" OR "Colombia" OR "Comoros" OR "Congo Democratic Republic" OR "Congo Republic" OR "Costa Rica" OR "Côte d'Ivoire" OR "Cuba" OR "Djibouti" OR "Dominica" OR "Dominican Republic" OR "Ecuador" OR "Egypt" OR "El Salvador" OR "Equatorial Guinea" OR "Eritrea" OR "Eswatini" OR "Ethiopia" OR "Fiji" OR "Gabon" OR "The Gambia" OR "Georgia" OR "Ghana" OR "Grenada" OR "Guatemala" OR "Guinea" OR "Guinea-Bissau" OR "Guyana" OR "Haiti" OR "Honduras" OR "India" OR "Indonesia" OR "Iran, Islamic Republic" OR "Iraq" OR "Jamaica" OR "Jordan" OR "Kazakhstan" OR "Kenya" OR "Kiribati" OR "Korea, Democratic People's Republic" OR "Kosovo" OR "Kyrgyz Republic" OR "Lao PDR" OR "Lebanon" OR "Lesotho" OR "Liberia" OR "Libya" OR "Madagascar" OR "Malawi" OR "Malaysia" OR "Maldives" OR "Mali" OR "Marshall Islands" OR "Mauritania" OR "Mexico" OR "Micronesia" OR "Moldova" OR "Mongolia" OR "Montenegro" OR "Morocco" OR "Mozambique" OR "Myanmar" OR "Namibia" OR "Nepal" OR "Nicaragua" OR "Niger" OR "Nigeria" OR "North Macedonia" OR "Pakistan" OR "Papua New Guinea" OR "Paraguay" OR "Peru" OR "Philippines" OR "Russian Federation" OR "Rwanda" OR "Samoa" OR "São Tomé and Principe" OR "Senegal" OR "Serbia" OR "Sierra Leone" OR "Solomon Islands" OR "Somalia" OR "South Africa" OR "South Sudan" OR "Sri Lanka" OR "St. Lucia" OR "St. Vincent and the Grenadines" OR "Sudan" OR "Suriname" OR "Syrian Arab Republic" OR "Tajikistan" OR "Tanzania" OR "Thailand" OR "Timor-Leste" OR "Togo" OR "Tonga" OR "Tunisia" OR "Turkey" OR "Turkmenistan" OR "Tuvalu" OR "Uganda" OR "Ukraine" OR "Uzbekistan" OR "Vanuatu" OR "Venezuela" OR "Vietnam" OR "West Bank and Gaza" OR "Yemen Republic" OR "Zambia" OR "Zimbabwe")

**PsycINFO**

Search date: 07 October 2022

Result: 803

ab(community based health insurance) OR ab(mutual health insurance) OR ab(micro health insurance) AND ab(("Afghanistan" OR "Albania" OR "Algeria" OR "American Samoa" OR "Angola" OR "Argentina" OR "Armenia" OR "Azerbaijan" OR "Bangladesh" OR "Belarus" OR "Belize" OR "Benin" OR "Bhutan" OR "Bolivia" OR "Bosnia and Herzegovina" OR "Botswana" OR "Brazil" OR "Bulgaria" OR "Burkina Faso" OR "Burundi" OR "Cabo Verde" OR "Cambodia" OR "Cameroon" OR "Central African Republic" OR "Chad" OR "China" OR "Colombia" OR "Comoros" OR "Congo Democratic Republic" OR "Congo Republic" OR "Costa Rica" OR "Côte d'Ivoire" OR "Cuba" OR "Djibouti" OR "Dominica" OR "Dominican Republic" OR "Ecuador" OR "Egypt" OR "El Salvador" OR "Equatorial Guinea" OR "Eritrea" OR "Eswatini" OR "Ethiopia" OR "Fiji" OR "Gabon" OR "The Gambia" OR "Georgia" OR "Ghana" OR "Grenada" OR "Guatemala" OR "Guinea" OR "Guinea-Bissau" OR "Guyana" OR "Haiti" OR "Honduras" OR "India" OR "Indonesia" OR "Iran, Islamic Republic" OR "Iraq" OR "Jamaica" OR "Jordan" OR "Kazakhstan" OR "Kenya" OR "Kiribati" OR "Korea, Democratic People's Republic" OR "Kosovo" OR "Kyrgyz Republic" OR "Lao PDR" OR "Lebanon" OR "Lesotho" OR "Liberia" OR "Libya" OR "Madagascar" OR "Malawi" OR "Malaysia" OR "Maldives" OR "Mali" OR "Marshall Islands" OR "Mauritania" OR "Mexico" OR "Micronesia" OR "Moldova" OR "Mongolia" OR "Montenegro" OR "Morocco" OR "Mozambique" OR "Myanmar" OR "Namibia" OR "Nepal" OR "Nicaragua" OR "Niger" OR "Nigeria" OR "North Macedonia" OR "Pakistan" OR "Papua New Guinea" OR "Paraguay" OR "Peru" OR "Philippines" OR "Russian Federation" OR "Rwanda" OR "Samoa" OR "São Tomé and Principe" OR "Senegal" OR "Serbia" OR "Sierra Leone" OR "Solomon Islands" OR "Somalia" OR "South Africa" OR "South Sudan" OR "Sri Lanka" OR "St. Lucia" OR "St. Vincent and the Grenadines" OR "Sudan" OR "Suriname" OR "Syrian Arab Republic" OR "Tajikistan" OR "Tanzania" OR "Thailand" OR "Timor-Leste" OR "Togo" OR "Tonga" OR "Tunisia" OR "Turkey" OR "Turkmenistan" OR "Tuvalu" OR "Uganda" OR "Ukraine" OR "Uzbekistan" OR "Vanuatu" OR "Venezuela" OR "Vietnam" OR "West Bank and Gaza" OR "Yemen Republic" OR "Zambia" OR "Zimbabwe"))

**Scopus**

Search date: 07 October 2022

Results: 726

( TITLE-ABS-KEY ( ( community AND based AND health AND insurance ) OR ( mutual AND health AND insurance ) OR ( micro AND health AND insurance ) OR ( prepayment AND scheme ) ) AND TITLE-ABS-KEY ( ( low AND middle AND income AND countr* ) OR ( developing AND countr* ) OR ( asia ) OR ( africa ) OR ( south AND america ) OR ( central AND america ) ) )

**Web of Science**

Search date: 08 October 2022

Results: 2,392

community based health insurance (Abstract) or mutual health insurance (Abstract) or micro health insurance (Abstract) or prepayment scheme (Abstract) and ("Afghanistan" OR "Albania" OR "Algeria" OR "American Samoa" OR "Angola" OR "Argentina" OR "Armenia" OR "Azerbaijan" OR "Bangladesh" OR "Belarus" OR "Belize" OR "Benin" OR "Bhutan" OR "Bolivia" OR "Bosnia and Herzegovina" OR "Botswana" OR "Brazil" OR "Bulgaria" OR "Burkina Faso" OR "Burundi" OR "Cabo Verde" OR "Cambodia" OR "Cameroon" OR "Central African Republic" OR "Chad" OR "China" OR "Colombia" OR "Comoros" OR "Congo Democratic Republic" OR "Congo Republic" OR "Costa Rica" OR "Côte d'Ivoire" OR "Cuba" OR "Djibouti" OR "Dominica" OR "Dominican Republic" OR "Ecuador" OR "Egypt" OR "El Salvador" OR "Equatorial Guinea" OR "Eritrea" OR "Eswatini" OR "Ethiopia" OR "Fiji" OR "Gabon" OR "The Gambia" OR "Georgia" OR "Ghana" OR "Grenada" OR "Guatemala" OR "Guinea" OR "Guinea-Bissau" OR "Guyana" OR "Haiti" OR "Honduras" OR "India" OR "Indonesia" OR "Iran, Islamic Republic" OR "Iraq" OR "Jamaica" OR "Jordan" OR "Kazakhstan" OR "Kenya" OR "Kiribati" OR "Korea, Democratic People's Republic" OR "Kosovo" OR "Kyrgyz Republic" OR "Lao PDR" OR "Lebanon" OR "Lesotho" OR "Liberia" OR "Libya" OR "Madagascar" OR "Malawi" OR "Malaysia" OR "Maldives" OR "Mali" OR "Marshall Islands" OR "Mauritania" OR "Mexico" OR "Micronesia" OR "Moldova" OR "Mongolia" OR "Montenegro" OR "Morocco" OR "Mozambique" OR "Myanmar" OR "Namibia" OR "Nepal" OR "Nicaragua" OR "Niger" OR "Nigeria" OR "North Macedonia" OR "Pakistan" OR "Papua New Guinea" OR "Paraguay" OR "Peru" OR "Philippines" OR "Russian Federation" OR "Rwanda" OR "Samoa" OR "São Tomé and Principe" OR "Senegal" OR "Serbia" OR "Sierra Leone" OR "Solomon Islands" OR "Somalia" OR "South Africa" OR "South Sudan" OR "Sri Lanka" OR "St. Lucia" OR "St. Vincent and the Grenadines" OR "Sudan" OR "Suriname" OR "Syrian Arab Republic" OR "Tajikistan" OR "Tanzania" OR "Thailand" OR "Timor-Leste" OR "Togo" OR "Tonga" OR "Tunisia" OR "Turkey" OR "Turkmenistan" OR "Tuvalu" OR "Uganda" OR "Ukraine" OR "Uzbekistan" OR "Vanuatu" OR "Venezuela" OR "Vietnam" OR "West Bank and Gaza" OR "Yemen Republic" OR "Zambia" OR "Zimbabwe") (Title)

**Global health library (currently known as Global Index Medicus)**

1. ***African Index Medicus (AIM)***

Search date: 09 October 2022

Result: 55

(Community based health insurance OR Mutual health insurance OR Micro health insurance)

1. ***Index Medicus for the Eastern Mediterranean Region (IMEAR)***

Search date: 09 October 2022

Result: 57

(Community based health insurance OR Mutual health insurance OR Micro health insurance)

1. ***Index Medicus for the South-East Asia Region (IMSEAR)***

Search date: 09 October 2022

Result: 72

(Community based health insurance OR Mutual health insurance OR Micro health insurance)

1. ***Latin America and the Caribbean Literature on Health Sciences / Literatura Latino-Americana e do Caribe em Ciências da Saúde (LILACS)***

Search date: 09 October 2022

Result: 860

(Community based health insurance OR Mutual health insurance OR Micro health insurance)

1. ***Western Pacific Region Index Medicus (WPRIM)***

Search date: 09 October 2022

Result: 441

(Community based health insurance OR Mutual health insurance OR Micro health insurance)
