# Supplementary material for: Impact of community-based health insurance in low- and middle-income countries: A systematic review and meta-analysis
Source: PLoS One. 2023 Jun 27;18(6):e0287600. doi: 10.1371/journal.pone.0287600 (PMC10298805; doi:10.1371/journal.pone.0287600)
Supplement: S1 Table — (DOCX) [file pone.0287600.s006.docx]

**S1 Table:** Inclusion criteria for studies reporting impact of community-based health insurance (CBHI) schemes on healthcare utilization and financial risk protection in low- and middle-income countries (LMICs)

|  | **Inclusion criteria** | **Exclusion criteria** |
| --- | --- | --- |
| **Population** | Individuals or households in low- and middle-income countries (LMIC) as defined by the World Bank. | High-income countries |
|  |  |  |
| **Intervention or Exposure** | Individuals or households enrolled in community-based health insurance (CBHI) schemes as defined by the World Health Organization, including mutual health insurance schemes, mutual health organizations, micro health insurance schemes, community-based prepayment schemes, and community health funds. | Individuals enrolled in social health insurance, private health insurance and national health insurance. |
|  |  |  |
| **Control or Comparison** | Individuals or households not enrolled in any insurance scheme | Individuals enrolled in social health insurance, private health insurance and national health insurance. |
|  |  |  |
| **Outcome(s)** | 1). Healthcare utilization and/or healthcare access  2). Financial risk protection assessed as out-of-pocket health expenditures; catastrophic health expenditures defined as a proportion of total household expenditure, household non-food expenditure, and/or capacity to pay of any threshold; or health payment-induced impoverishments including sale of household assets, borrowing, etc. | Health costs that are not clearly defined, or reported as multidimensional poverty index (MPI) score, or similar terms  Non-health- or health-related-payment induced poverty |
|  |  |  |
| **Study** | A). Randomized controlled (field) trials (RCTs)  B). Quasi-randomized / non-randomized controlled trials or studies including;  ** Studies where methods of allocating are not random but are intended to produce similar groupings of treatment and control including propensity score matching methods and regression discontinuity design.  ** Controlled before-and-after (CBA) studies or difference-in-difference (DID) studies; if the pre- and post-intervention periods for the study and control groups are the same and the controls are matched  ** Regression studies where probability of selection into treatment is considered through instrumental variables  C). Population-based cross-sectional studies in which endogeneity in health insurance choices due to individuals self-selecting into or out of CBHI schemes is addressed using Bayesian adjustment | Case reports, case series, reviews, letters to editors, commentary, study protocols, and baseline assessment reports  Interviews with programme managers, hospital administrators, insurance managers, etc.  Studies reporting impact measures using regression or matching. |
|  |  | Articles reporting sample data already included in the review |
|  | *Publication status*: published, preprints, and grey literature (unpublished) |  |
|  | *Availability*: Full study available | Conference abstracts, Poster presentations, Retracted studies |
|  |  |  |
