# Supplementary material for: Impact of community-based health insurance in low- and middle-income countries: A systematic review and meta-analysis
Source: PLoS One. 2023 Jun 27;18(6):e0287600. doi: 10.1371/journal.pone.0287600 (PMC10298805; doi:10.1371/journal.pone.0287600)
Supplement: S2 Table — (DOCX) [file pone.0287600.s007.docx]

**S2 Table:** Retrieved full text articles/studies excluded from the review and reasons for exclusion

| **SN** | **Study** | **Reason(s) for exclusion** |
| --- | --- | --- |
| 1 | Acharya & Ranson 2005 [1] | Review |
| 2 | Adinma et al. 2010 [2] | Data already included in Review >>> Adinma et al. 2011 |
| 3 | Ahmed et al. 2021 [3] | Identification strategy not eligible |
| 4 | Alatinga & Fielmua 2011 [4] | Identification strategy not eligible |
| 5 | Alula et al. 2021 [5] | Data already included in Review >>> Alemayehu et al. 2022 |
| 6 | Asfaw and Braun 2004 [6] | Identification strategy not eligible |
| 7 | Atim & Sock 2000 [7] | Identification strategy not eligible |
| 8 | Atim 1999 [8] | Identification strategy not eligible |
| 9 | Atnafu & Gebremedhin 2020 [9] | Identification strategy not eligible |
| 10 | Bennet et al. 1998 [10] | Review |
| 11 | Bhat et al. 2017 [11] | Identification strategy not eligible |
| 12 | Blanchard-Horan 2007 [12] | Identification strategy not eligible |
| 13 | Broomberg & Price 1990 [13] | Identification strategy not eligible |
| 14 | Carrin et al. 1999 [14] | Identification strategy not eligible |
| 15 | Chee et al. 2002 [15] | Identification strategy not eligible |
| 16 | Cheema et al. 2020 [16] | Identification strategy not eligible |
| 17 | Criel & Kegel 1997 [17] | Identification strategy not eligible |
| 18 | Criel et al. 1999 [18] | Identification strategy not eligible |
| 19 | Dekker & Wilms 2010 [19] | Identification strategy not eligible |
| 20 | Desai et al. 2011 [20] | Identification strategy not eligible |
| 21 | Devadasan et al. 2004 [21] | Identification strategy not eligible |
| 22 | Devadasan et al. 2007 [22] | Identification strategy not eligible |
| 23 | Devadasan et al. 2010 [23] | Identification strategy not eligible |
| 24 | Diop et al. 2006 [24] | Data already included in Review >>> Chankova et al. 2008 |
| 25 | Dror et al. 2005 [25] | Identification strategy not eligible |
| 26 | Dror et al. 2009 [26] | Identification strategy not eligible |
| 27 | Ethiopia Health Insurance Agency 2015 [27] | Identification strategy not eligible |
| 28 | Ekman 2007 [28] | Identification strategy not eligible |
| 29 | Fakunle et al. 2014 [29] | Identification strategy not eligible |
| 30 | Fischer et al. 2018 [30] | Not CBHI |
| 31 | Fitzpatrick & Thornton 2019 [31] | Not CBHI |
| 32 | Franco et al. 2008 [32] | Identification strategy not eligible |
| 33 | Galarraga et al. 2010 [33] | Not CBHI |
| 34 | Geng et al. 2018 [34] | Identification strategy not eligible |
| 35 | Haddad et al. 2012 [35] | Identification strategy not eligible |
| 36 | Hamid et al. 2011 [36] | Identification strategy not eligible |
| 37 | Haushofer et al. 2020 [37] | Not CBHI |
| 38 | Hou et al. 2014 [38] | Identification strategy not eligible |
| 39 | Hounton et al. 2012 [39] | Identification strategy not eligible |
| 40 | Idris et al. 2017 [40] | Not CBHI |
| 41 | Islam et al. 2012 [41] | Identification strategy not eligible |
| 42 | Jakab et al. 2001 [42] | Identification strategy not eligible |
| 43 | James et al. 2008 [43] | Identification strategy not eligible |
| 44 | Jembere 2018 (A) [44] | Identification strategy not eligible |
| 45 | Jembere 2018 (B) [45] | Identification strategy not eligible |
| 46 | Joshi et al. 2020 [46] | Identification strategy not eligible |
| 47 | Jutting 2001 [47] | Data already included in Review >>> Jutting 2004 |
| 48 | Kassie & Tefera 2019 [48] | Identification strategy not eligible |
| 49 | Koch et al. 2022 [49] | Identification strategy not eligible |
| 50 | Kuwekita et al. 2015 (FRENCH) [50] | Identification strategy not eligible |
| 51 | Lakshmi 2019 [51] | Identification strategy not eligible |
| 52 | Liu et al. 2019 [52] | Identification strategy not eligible |
| 53 | Lu et al. 2017 [53] | Identification strategy not eligible |
| 54 | Mahmud & Hilton 2020 [54] | Not CBHI |
| 55 | McFarlane & Sammon 2000 [55] | Case study |
| 56 | Mebratie 2015 (Thesis) [56] | Data already included in Review >>> Mebratie et al. 2019 |
| 57 | Mebratie et al. 2014 [57] | Data already included in Review >>> Mebratie et al. 2019 |
| 58 | Msuya et al. 2004 [58] | Identification strategy not eligible |
| 59 | Msuya et al. 2007 [59] | Identification strategy not eligible |
| 60 | Musango et al. 2004 (FRENCH) [60] | Identification strategy not eligible |
| 61 | Mwaura & Pongpanich 2012 [61] | Identification strategy not eligible |
| 62 | Ndongo et al. 2014 (FRENCH) [62] | Identification strategy not eligible |
| 63 | Okonofua et al. 2022 [63] | Could not extract the impact of CBHI |
| 64 | Parmar et al. 2014 [64] | Identification strategy not eligible |
| 65 | Peterson et al. 2015 [65] | Identification strategy not eligible |
| 66 | Peterson et al. 2018 [66] | Identification strategy not eligible |
| 67 | Ranson 2001 [67] | Identification strategy not eligible |
| 68 | Rao et al. 2011 [68] | Identification strategy not eligible |
| 69 | Richard 2005 (FRENCH) [69] | Review |
| 70 | Ron 1999 [70] | Review |
| 71 | Rukundo 2018 (Thesis) [71] | Data already included in Review >>> Nshakira-Rukundo et al. 2021 |
| 72 | Saksena et al. 2011 [72] | Identification strategy not eligible |
| 73 | Schneider & Diop 2001 [73] | Identification strategy not eligible |
| 74 | Schneider & Hanson 2005 [74] | Identification strategy not eligible |
| 75 | Sheth 2014 [75] | Data already included in Review >>> Sheth 2021 |
| 76 | Smith & Sulzbach 2008 [76] | Identification strategy not eligible |
| 77 | Stoermer et al. 2012 [77] | Identification strategy not eligible |
| 78 | Sudha 2006 [78] | Review |
| 79 | Tanko et al. 2015 [79] | Identification strategy not eligible |
| 80 | Tesfay 2014 (Thesis) [80] | Identification strategy not eligible |
| 81 | The World Bank 2010 [81] | Data already included in Review >>> Alkenbrack & Lindelow 2015 |
| 82 | Wagstaff et al. 2007 [82] | Data already included in Review >>> Wagstaff et al. 2009 |
| 83 | Werner 2009 [83] | Case study |
| 84 | Woldemichael et al. 2016 [84] | Data already included in Review >>> Woldemichael et al. 2019 |
| 85 | Yi et al. 2009 [85] | Identification strategy not eligible |
| 86 | Yip & Hsiao 2009 [86] | Identification strategy not eligible |
| 87 | You & Kobayashi 2011 [87] | Identification strategy not eligible |
| 88 | Zhou et al. 2009 [88] | Identification strategy not eligible |
