# Supplementary material for: Impact of community-based health insurance in low- and middle-income countries: A systematic review and meta-analysis
Source: PLoS One. 2023 Jun 27;18(6):e0287600. doi: 10.1371/journal.pone.0287600 (PMC10298805; doi:10.1371/journal.pone.0287600)
Supplement: S3 Table — (DOCX) [file pone.0287600.s008.docx]

**S3 Table:** Sub-group analysis of the impact of CBHI on healthcare utilization (non-specific) in LMICs

| **Sub-groups** | **Number of studies** | **Sample size** | **Odds ratio**  **(95% CI)** | ***p-value***** | ***I*^2^** |
| --- | --- | --- | --- | --- | --- |
| **Overall pooled estimate** | 4 | 5,122 | 1.60 (1.04 – 2.47) |  | 85.1% |
|  |  |  |  |  |  |
| **CBHI model** |  |  |  | 0.204 |  |
| - Provider-based | 0 |  |  |  |  |
| - Community-driven and community-managed | 3 | 4,470 | 1.45 (0.90 – 2.32) |  | 81.5% |
| - Government-supported community-involved | 1 | 652 | 2.16 (1.45 – 3.07) |  | -- |
|  |  |  |  |  |  |
| **World Bank region** |  |  |  | 0.228 |  |
| - East Asia & Pacific | 0 |  |  |  |  |
| - South Asia | 1 | 1,292 | 2.11 (1.45 – 3.07) |  | -- |
| - Sub-Saharan Africa | 3 | 3,830 | 1.45 (0.90 – 2.34) |  | 80.4% |
|  |  |  |  |  |  |
| **Income status** |  |  |  | 0.610 |  |
| - Low income | 2 | 3,486 | 1.86 (1.20 – 2.88) |  | 26.2% |
| - Lower middle-income | 2 | 1,636 | 1.55 (0.91 – 2.67) |  | 87.3% |
| - Upper middle-income | 0 |  |  |  |  |
|  |  |  |  |  |  |
| **Study design** |  |  |  | <0.001 |  |
| - Randomized controlled trials (RCT) | 2 | 3,178 | 1.14 (1.06 – 1.32) |  | 0.0% |
| - Non-RCT and Quasi-experimental | 2 | 1,944 | 2.13 (1.62 – 2.80) |  | 0.0% |
|  |  |  |  |  |  |
| **Publication status** |  |  |  | NA |  |
| - Non-peer reviewed | 0 |  |  |  |  |
| - Peer reviewed | 4 | 5,122 | 1.60 (1.04 – 2.47) |  | 85.1% |
|  |  |  |  |  |  |
| **Study quality** |  |  |  | 0.204 |  |
| - Low risk of bias | 3 | 4,470 | 1.45 (0.90 – 2.32) |  | 81.5% |
| - Some concerns or high risk of bias | 1 | 652 | 2.16 (1.45 – 3.22) |  | -- |

** P-value for the test of group differences. CI: Confidence interval. NA: Not applicable
