# Supplementary material for: Impact of community-based health insurance in low- and middle-income countries: A systematic review and meta-analysis
Source: PLoS One. 2023 Jun 27;18(6):e0287600. doi: 10.1371/journal.pone.0287600 (PMC10298805; doi:10.1371/journal.pone.0287600)
Supplement: S4 Table — (DOCX) [file pone.0287600.s009.docx]

**S4 Table**: Sub-group analysis of the impact of CBHI on outpatient health services in LMICs.

| **Sub-groups** | **Number of studies** | **Sample size** | **Odds ratio**  **(95% CI)** | ***p-value***** | ***I*^2^** |
| --- | --- | --- | --- | --- | --- |
| **Healthcare utilization: Overall pooled estimate** | 10 | 42,210 | 1.69 (1.32 – 2.17) |  | 87.9% |
|  |  |  |  |  |  |
| **CBHI model** |  |  |  | 0.933 |  |
| - Provider-based | 1 | 1,806 | 1.81 (1.10 – 2.98) |  | -- |
| - Community-driven and community-managed | 3 | 5,048 | 1.76 (0.81 – 3.85) |  | 89.5% |
| - Government-supported community-involved | 6 | 35,356 | 1.63 (1.23 – 2.18) |  | 89.1% |
|  |  |  |  |  |  |
| **World Bank region** |  |  |  | 0.002 |  |
| - East Asia & Pacific | 1 | 3,952 | 1.01 (0.81 – 1.27) |  | -- |
| - South Asia | 0 |  |  |  |  |
| - Sub-Saharan Africa | 9 | 38,258 | 1.82 (1.36 – 2.41) |  | 88.5% |
|  |  |  |  |  |  |
| **Income status** |  |  |  | 0.004 |  |
| - Low income | 6 | 22,618 | 1.95 (1.28 – 2.96) |  | 92.4% |
| - Lower middle-income | 3 | 15,640 | 1.55 (1.27 – 1.91) |  | 0.0% |
| - Upper middle-income | 1 | 3,952 | 1.01 (0.81 – 1.26) |  | -- |
|  |  |  |  |  |  |
| **Study design** |  |  |  | <0.001 |  |
| - Randomized controlled trials (RCT) | 1 | 1,309 | 3.99 (2.53 – 6.27) |  | -- |
| - Non-RCT and Quasi-experimental | 9 | 40,901 | 1.55 (1.24 – 1.94) |  | 84.1% |
|  |  |  |  |  |  |
| **Publication status** |  |  |  | 0.006 |  |
| - Non-peer reviewed | 1 | 8,384 | 1.79 (1.34 – 2.39) |  | -- |
| - Peer reviewed | 9 | 33,826 | 1.18 (1.10 – 1.26) |  | 83.8% |
|  |  |  |  |  |  |
| **Study quality** |  |  |  | 0.947 |  |
| - Low risk of bias | 6 | 36,255 | 1.71 (1.24 – 2.33) |  | 91.2% |
| - Some concerns or high risk of bias | 4 | 5,955 | 1.67 (1.06 – 2.63) |  | 74.8% |

** P-value for the test of group differences. CI: Confidence interval. NA: Not applicable
