# Supplementary material for: Impact of community-based health insurance in low- and middle-income countries: A systematic review and meta-analysis
Source: PLoS One. 2023 Jun 27;18(6):e0287600. doi: 10.1371/journal.pone.0287600 (PMC10298805; doi:10.1371/journal.pone.0287600)
Supplement: S5 Table — (DOCX) [file pone.0287600.s010.docx]

**S5 Table**: Sub-group analysis of the impact of CBHI on OOP health expenditure in LMICs.

| **Sub-groups** | **Number of studies** | **Sample size** | **Odds ratio**  **(95% CI)** | ***p-value***** | ***I*^2^** |
| --- | --- | --- | --- | --- | --- |
| **Healthcare utilization: Overall pooled estimate** | 4 | 8,983 | 0.94 (0.92 – 0.97) |  | 87.9% |
|  |  |  |  |  |  |
| **CBHI model** |  |  |  | 0.630 |  |
| - Provider-based | 0 |  |  |  |  |
| - Community-driven and community-managed | 3 | 5,031 | 0.97 (0.87 – 1.07) |  | 0.0% |
| - Government-supported community-involved | 1 | 3,952 | 1.13 (0.59 – 2.17) |  | -- |
|  |  |  |  |  |  |
| **World Bank region** |  |  |  | 0.380 |  |
| - East Asia & Pacific | 1 | 3,952 | 1.09 (0.89 – 1.34) |  | -- |
| - South Asia | 1 | 1,292 | 0.94 (0.91 – 0.96) |  | -- |
| - Sub-Saharan Africa | 2 | 3,739 | 1.07 (0.87 – 1.32) |  | 0.2% |
|  |  |  |  |  |  |
| **Income status** |  |  |  | 0.296 |  |
| - Low income | 1 | 2,659 | 1.09 (0.89 – 1.34) |  | -- |
| - Lower middle-income | 2 | 2,372 | 0.94 (0.91 – 0.96) |  | 25.4% |
| - Upper middle-income | 1 | 3,952 | 1.13 (0.59 – 2.17) |  | -- |
|  |  |  |  |  |  |
| **Study design** |  |  |  | NA |  |
| - Randomized controlled trials (RCT) | 0 |  |  |  |  |
| - Non-RCT and Quasi-experimental | 4 | 8,983 | 0.94 (0.92 – 0.97) |  | 0.1% |
|  |  |  |  |  |  |
| **Publication status** |  |  |  | NA |  |
| - Non-peer reviewed | 0 |  |  |  |  |
| - Peer reviewed | 4 | 8,983 | 0.94 (0.92 – 0.97) |  | 0.1% |
|  |  |  |  |  |  |
| **Study quality** |  |  |  | 0.205 |  |
| - Low risk of bias | 2 | 3,739 | 0.94 (0.91 – 0.96) |  | 0.0% |
| - Some concerns or high risk of bias | 2 | 5,244 | 1.07 (0.87 – 1.32) |  | 0.2% |

** P-value for the test of group differences. CI: Confidence interval. NA: Not applicable
