# Supplementary material for: Impact of community-based health insurance in low- and middle-income countries: A systematic review and meta-analysis
Source: PLoS One. 2023 Jun 27;18(6):e0287600. doi: 10.1371/journal.pone.0287600 (PMC10298805; doi:10.1371/journal.pone.0287600)
Supplement: S6 Table — (DOCX) [file pone.0287600.s011.docx]

**S6 Table**: Sub-group analysis of the impact of CBHI on catastrophic health expenditure at 10% total household expenditure threshold in LMICs.

| **Sub-groups** | **Number of studies** | **Sample size** | **Odds ratio**  **(95% CI)** | ***p-value***** | ***I*^2^** |
| --- | --- | --- | --- | --- | --- |
| **Healthcare utilization: Overall pooled estimate** | 4 | 10,614 | 0.69 (0.54 – 0.88) |  | 59.6% |
|  |  |  |  |  |  |
| **CBHI model** |  |  |  | 0.309 |  |
| - Provider-based | 0 |  |  |  |  |
| - Community-driven and community-managed | 1 | 1,530 | 0.83 (0.58 – 1.19) |  | -- |
| - Government-supported community-involved | 3 | 9,084 | 0.65 (0.48 – 0.88) |  | 66.2% |
|  |  |  |  |  |  |
| **World Bank region** |  |  |  | 0.606 |  |
| - East Asia & Pacific | 2 | 4,846 | 0.74 (0.56 – 0.98) |  | 40.1% |
| - South Asia | 0 |  |  |  |  |
| - Sub-Saharan Africa | 2 | 5,768 | 0.64 (0.39 – 1.04) |  | 77.6% |
|  |  |  |  |  |  |
| **Income status** |  |  |  | 0.376 |  |
| - Low income | 2 | 5,768 | 0.64 (0.39 – 1.04) |  | 77.6% |
| - Lower middle-income | 1 | 3,000 | 0.65 (0.48 – 0.87) |  | -- |
| - Upper middle-income | 1 | 1,846 | 0.86 (0.63 – 1.17) |  | -- |
|  |  |  |  |  |  |
| **Study design** |  |  |  | 0.309 |  |
| - Randomized controlled trials (RCT) | 1 | 1,530 | 0.83 (0.58 – 1.19) |  | -- |
| - Non-RCT and Quasi-experimental | 3 | 9,084 | 0.69 (0.54 – 0.88) |  | 66.2% |
|  |  |  |  |  |  |
| **Publication status** |  |  |  | NA |  |
| - Non-peer reviewed | 0 |  |  |  |  |
| - Peer reviewed | 4 | 10,614 | 0.69 (0.54 – 0.88) |  | 59.6% |
|  |  |  |  |  |  |
| **Study quality** |  |  |  | NA |  |
| - Low risk of bias | 4 | 10,614 | 0.69 (0.54 – 0.88) |  | 59.6% |
| - Some concerns or high risk of bias | 0 |  |  |  |  |

** P-value for the test of group differences. CI: Confidence interval. NA: Not applicable
