# Supplementary material for: Impact of community-based health insurance in low- and middle-income countries: A systematic review and meta-analysis
Source: PLoS One. 2023 Jun 27;18(6):e0287600. doi: 10.1371/journal.pone.0287600 (PMC10298805; doi:10.1371/journal.pone.0287600)
Supplement: S7 Table — (DOCX) [file pone.0287600.s012.docx]

**S7 Table**: Sub-group analysis of the impact of CBHI on catastrophic health expenditure at 40% non-food expenditure threshold in LMICs.

| **Sub-groups** | **Number of studies** | **Sample size** | **Odds ratio**  **(95% CI)** | ***p-value***** | ***I*^2^** |
| --- | --- | --- | --- | --- | --- |
| **Healthcare utilization: Overall pooled estimate** | 4 | 22,543 | 0.72 (0.54 – 0.96) |  | 76.6% |
|  |  |  |  |  |  |
| **CBHI model** |  |  |  | NA |  |
| - Provider-based | 0 |  |  |  |  |
| - Community-driven and community-managed | 0 |  |  |  |  |
| - Government-supported community-involved | 4 | 22,543 | 0.72 (0.54 – 0.96) |  | 76.6% |
|  |  |  |  |  |  |
| **World Bank region** |  |  |  | 0.092 |  |
| - East Asia & Pacific | 2 | 11,405 | 0.91 (0.78 – 1.06) |  | 0.0% |
| - South Asia | 0 |  |  |  |  |
| - Sub-Saharan Africa | 2 | 11,138 | 0.52 (0.28 – 0.97) |  | 78.2% |
|  |  |  |  |  |  |
| **Income status** |  |  |  | 0.241 |  |
| - Low income | 2 | 11,138 | 0.52 (0.28 – 0.97) |  | 78.2% |
| - Lower middle-income | 1 | 8,304 | 0.90 (0.65 – 1.25) |  | -- |
| - Upper middle-income | 1 | 3,101 | 0.91 (0.76 – 1.08) |  | -- |
|  |  |  |  |  |  |
| **Study design** |  |  |  | NA |  |
| - Randomized controlled trials (RCT) | 0 |  |  |  |  |
| - Non-RCT and Quasi-experimental | 4 | 22,543 | 0.72 (0.54 – 0.96) |  | 76.6% |
|  |  |  |  |  |  |
| **Publication status** |  |  |  | 0.523 |  |
| - Non-peer reviewed | 2 | 12,542 | 0.58 (0.24 – 1.44) |  | 87.3% |
| - Peer reviewed | 2 | 10,001 | 0.79 (0.60 – 1.05) |  | 76.3% |
|  |  |  |  |  |  |
| **Study quality** |  |  |  | 0.230 |  |
| - Low risk of bias | 3 | 14,239 | 0.66 (0.46 – 0.96) |  | 83.5% |
| - Some concerns or high risk of bias | 1 | 8,304 | 0.90 (0.54 – 1.25) |  | -- |

** P-value for the test of group differences. CI: Confidence interval. NA: Not applicable
