# Supplementary material for: Impact of community-based health insurance in low- and middle-income countries: A systematic review and meta-analysis
Source: PLoS One. 2023 Jun 27;18(6):e0287600. doi: 10.1371/journal.pone.0287600 (PMC10298805; doi:10.1371/journal.pone.0287600)
Supplement: S8 Table — (PDF) [file pone.0287600.s013.pdf]

**Author(s):** Paul Eze, Stanley Ilechukwu, and Lucky Osaheni Lawani
**Question:** Impact of community-based health insurance compared to uninsured in low- and middle-income countries?
**Setting:** Low- and middle-income countries (LMICs)
**Bibliography:**

| Certainty assessment | | | | | | | № of patients | | Effect | | Certainty | Importance |
|---|---|---|---|---|---|---|---|---|---|---|---|---|
| № of studies | Study design | Risk of bias | Inconsistency | Indirectness | Imprecision | Other considerations | Community-based health insurance | uninsured | Relative (95% CI) | Absolute (95% CI) | | |
| **Impact of CBHI on healthcare utilization (non-specific)** | | | | | | | | | | | | |
| 2 | randomised trials | not serious | not serious | not serious | not serious | strong association all plausible residual confounding would reduce the demonstrated effect dose response gradient | | | **OR 1.214** (1.092 to 1.349) | **1 fewer per 1,000** (from 1 fewer to 1 fewer) | ⊕⊕⊕⊕ High | CRITICAL |
| **Impact of CBHI on healthcare utilization (non-specific)** | | | | | | | | | | | | |
| 2 | observational studies | not serious | not serious | not serious | not serious | very strong association all plausible residual confounding would reduce the demonstrated effect dose response gradient | | | **OR 2.134** (1.624 to 2.804) | **2 fewer per 1,000** (from 3 fewer to 2 fewer) | ⊕⊕⊕⊕ High | CRITICAL |
| **Impact of CBHI on use of outpatient services** | | | | | | | | | | | | |
| 1 | randomised trials | not serious | not serious | not serious | not serious | very strong association | | | **OR 3.985** (2.474 to 6.132) | **4 fewer per 1,000** (from 6 fewer to 2 fewer) | ⊕⊕⊕⊕ High | CRITICAL |
| **Impact of CBHI on use of outpatient services** | | | | | | | | | | | | |
| 6 | observational studies | not serious | serious[a] | not serious | not serious | all plausible residual confounding would reduce the demonstrated effect | | | **OR 1.549** (1.238 to 1.938) | **2 fewer per 1,000** (from 2 fewer to 1 fewer) | ⊕⊕◯◯ Low | CRITICAL |
| **Impact of CBHI on Inpatient hospitalization** | | | | | | | | | | | | |
| 2 | observational studies | not serious | serious[b] | not serious | not serious | all plausible residual confounding would reduce the demonstrated effect | | | **OR 1.53** (0.74 to 3.14) | **2 fewer per 1,000** (from 3 fewer to 1 fewer) | ⊕⊕◯◯ Low | CRITICAL |
| **Impact of CBHI on Health facility delivery** | | | | | | | | | | | | |
| 2 | observational studies | not serious | not serious | not serious | not serious | all plausible residual confounding would reduce the demonstrated effect | | | **OR 2.21** (1.61 to 3.02) | **2 fewer per 1,000** (from 3 fewer to 2 fewer) | ⊕⊕⊕⊕ High | CRITICAL |
| **Impact of CBHI on OOP expenditure** | | | | | | | | | | | | |
| 4 | observational studies | not serious | not serious | not serious | not serious | all plausible residual confounding would reduce the demonstrated effect | | | **OR 0.94** (0.92 to 0.97) | **1 fewer per 1,000** (from 1 fewer to 1 fewer) | ⊕⊕⊕⊕ High | CRITICAL |
| **Impact of CBHI on catastrophic health expenditure at 10% total household expenditure** | | | | | | | | | | | | |
| 1 | randomised trials | not serious | not serious | not serious | not serious | all plausible residual confounding would reduce the demonstrated effect | | | **OR 0.830** (0.546 to 1.114) | **1 fewer per 1,000** (from 1 fewer to 1 fewer) | ⊕⊕⊕⊕ High | CRITICAL |
| **Impact of CBHI on catastrophic health expenditure at 10% total household expenditure** | | | | | | | | | | | | |
| 3 | observational studies | not serious | not serious | not serious | not serious | all plausible residual confounding would reduce the demonstrated effect dose response gradient | | | **OR 0.651** (0.482 to 0.880) | **1 fewer per 1,000** (from 1 fewer to 0 fewer) | ⊕⊕⊕⊕ High | CRITICAL |
| **Impact of CBHI on catastrophic health expenditure at 40% non-food expenditure** | | | | | | | | | | | | |
| 4 | observational studies | not serious | not serious | not serious | not serious | all plausible residual confounding would reduce the demonstrated effect dose response gradient | | | **OR 0.722** (0.543 to 0.962) | **1 fewer per 1,000** (from 1 fewer to 1 fewer) | ⊕⊕⊕⊕ High | CRITICAL |

**CI:** confidence interval; **OR:** odds ratio

**Explanations**

a. High heterogeneity as evidenced by the I2 = 87.9%
b. High heterogeneity as evidenced by the I2 = 82.3%
